# Trends in Pneumonia and Influenza-associated Hospitalizations in South Korea, 2002-2005

**DOI:** 10.3329/jhpn.v29i6.9894

**Published:** 2011-12

**Authors:** Soon Ae Kim, Paul E. Kilgore, Sang-Yi Lee, Batmunkh Nyambat, Moran Ki

**Affiliations:** ^1^Division of Translational Research, International Vaccine Institute, Seoul, South Korea (Present affiliation: Department of Pharmacy Practice, Eugene Applebaum College of Pharmacy and Health Sciences, Wayne State University, Detroit, Michigan, USA); ^2^Korean National Health Insurance Corporation, Seoul, South Korea (Present affiliation: Department of Health Policy and Management, School of Medicine, Cheju National University, Jeju, South Korea); ^3^Department of Preventive Medicine, Eulji University College of Medicine, Daejeon, South Korea

**Keywords:** Hospitalization, ICD-10 codes, Influenza, National health insurance, Pneumonia, South Korea

## Abstract

Pneumonia and influenza are leading causes of morbidity and mortality across the globe. Korea has established the national health-insurance system to cover the entire Korean population since 1989. The aim of this study was to describe the epidemiologic trends in pneumonia and influenza-associated hospitalizations and deaths using the Korean National Health Insurance databases and national vital statistics. During 2002-2005, 989,472 hospitalizations and 10,543 deaths due to pneumonia and influenza were recorded. Eighty-one percent of the hospitalizations were related to diagnoses with unspecified aetiology. The average annual rate of hospitalizations due to pneumonia and influenza was 5.2 per 1,000 people [95% confidence interval (CI) 5.2-5.3], and the hospitalization rate increased by 28% (from 4.5 to 5.8 per 1,000 people) during the four-year study period. In addition, deaths due to pneumonia and influenza increased by 48% (2,829 during 2003, 3,522 during 2004, and 4,192 during 2005). Overall, the national burden of hospitalizations and deaths due to pneumonia and influenza in Korea was high, and it increased for all age-groups during the study period. A comprehensive review of potential interventions by the government authorities should aim to reduce the burden of pneumonia and influenza.

## INTRODUCTION

Globally, pneumonia and influenza are leading causes of morbidity and mortality (1,2). In 2005, the United Nations Children's Fund (UNICEF) and the World Health Organization (WHO) reported that an estimated 1.6 million people die of pneumococcal disease every year, including 700,000 to one million children aged less than five years (2). More than 70% of patients with pneumonia were found in Africa and Asia (3). Each year, an estimated 5-10% of adults and 20-30% of children become ill due to influenza. In addition, 250,000-500,000 deaths and 3-5 million severe influenza cases, which occur each year, are associated with seasonal epidemics of influenza (4). In the USA, there are more than 1.6 million annual hospitalizations attributable to pneumonia and influenza, of which 65% have pneumonia or influenza as the primary diagnosis (5-7). Each year in the USA, over 36,000 deaths are attributable to pneumonia and influenza.

With growing recognition of the impact of pneumonia and influenza, prevention through vaccination has been recommended for both children and adults but the vaccine uptake has been low in resource-limited countries (8,9). While available vaccines have been shown to reduce the burden of pneumonia and influenza, the choice of antimicrobial therapy is likely to diminish case fatality, duration of hospitalization, and the frequency of clinical sequelae (10-13). In South Korea, the Health Institute for Review and Assessment Service (HIRA) and the National Health Insurance Corporation (NHIC) reported that organism-unspecified pneumonia was the fifth leading cause of inpatient health-insurance claims during 2005 (14). However, as of 2010, limited epidemiologic information regarding the recent trends in pneumonia and influenza in Korea is available.

In this study, we describe the burden of hospitalizations due to pneumonia and influenza and their epidemiologic trends using national health-insurance and national mortality databases. Results of this analysis will be useful to support the development of improved public-health policies for the control of pneumonia and influenza in Korea.

## MATERIALS AND METHODS

The National Health Insurance Programme reimburses providers for medical services, and the national health-insurance system covers 97% of the Korean population through mandatory contributions from insured residents. An additional 3% of the Korean population is covered through the Korean Medical Aid Programme which provides healthcare to low-income households (14,15).

We accessed the national health-insurance databases from January 2002 through December 2005, which contain the patient's date of birth, sex, *International Classification of Diseases*, 10^th^ Revision (ICD-10) codes for primary and secondary discharge diagnoses, dates of admission and discharge, type of hospital (primary, secondary, or tertiary), and the patient's address. Medical institutions receive medical service-fees from the Government by issuing reimbursement claims every month. For any patient with multiple hospital admissions, records were combined and considered the same disease episode if the date of admission was less than 30 days following the most recent date of discharge. To ensure confidentiality of patient information during analysis, individual national registration numbers were replaced with an unrelated unique eight-digit identification number assigned by the data-management staff of the Health Policy Research Institutes, NHIC.

For this analysis, a hospitalization was defined by admission to an inpatient facility for 24 hoursor more. Records of hospitalizations due to pneumonia and influenza were included for analysis, if the ICD-10 diagnostic codes for pneumonia and influenza were listed for either the primary or the secondary diagnosis. The ICD-10 codes are a valid method for the diagnosis of pneumonia in a previous study (16). The diagnostic codes were classified based on unspecified (i.e. a clinical syndrome with no mention of pathogen) and specific diagnoses (i.e. the pathogen is mentioned in diagnosis). We grouped the following diagnoses with unspecified aetiology as presumed infectious and non-infectious pneumonia (B05.2, J12.8 to J12.9, J15.8 to J15.9, J16.8, J17.0 to J17.3, J178, J18.0 to J18.1, J18.8 to J18.9, and J18.2) and bronchitis (J20.0, J20.1). Aetiology-specific causes included in this analysis are as follows: *Haemophilus influenzae*-associated pneumonia (J14), influenza (J10.0 to J10.1, J11.0, J11.1, J10.8, and J11.8), *Mycoplasma pneumoniae* (J15.7), *Streptococcus pneumoniae*-asscoated pneumonia (J13), other bacterial pneumonias (A54.8, J15.0 to J15.6, and J16.0), and other viral pneumonias (B012, and J12.0 to J12.2). We also accessed the national vital statistics from the Korea National Statistical Office (http://www.kosis.kr) to describe the patterns of deaths for the 2003-2005 period. Using the same list of pneumonia and influenza ICD-10 diagnostic codes applied in the analysis of hospitalization records, we identified deaths due to pneumonia and influenza by analyzing the primary and secondary causes of death.

In Korea, the Government conducts a national census every five years. Projected population data are available for years that occur during the intercensus periods. The government census office performs population projection estimates based on births, deaths, and immigrations using cohort component methods (www.nso.go.kr). To calculate the annual and age-specific rates of hospitalizations, national population projections for 2002 through 2004 were used as denominators. We also adjusted for hospitalization rate using the national census data of 2005 during the study period.

Analysis of data was performed using the SAS statistical software (version 9.12) (SAS Institute, Inc., Cary, North Carolina, USA) and the Stata software (version 11.0) (StataCorp LP, Texas, TX, USA).

## RESULTS

### General characteristics and trends by age-group and diagnosis

During 2002-2005, there were 21,781,400 all-cause hospitalizations among all age-groups in Korea. This total included 4,753,009 hospitalizations during 2002, 5,399,067 during 2003, 5,706,573 during 2004, and 5,922,751 during 2005. During the four-year study period, we identified 989,472 hospitalizations due to pneumonia and influenza among all age-groups in Korea. The proportion of hospitalizations due to pneumonia and influenza ranged from 4.5% to 4.6% during 2002-2005, and the proportion of deaths associated with pneumonia and influenza ranged from 1.1% to 1.7% of all deaths during 2003-2005 (Table 1). Of all the hospitalizations, we found that 81.1% of patients (n=802,462) were admitted to tertiary-care hospitals.

**Fig. 1.**
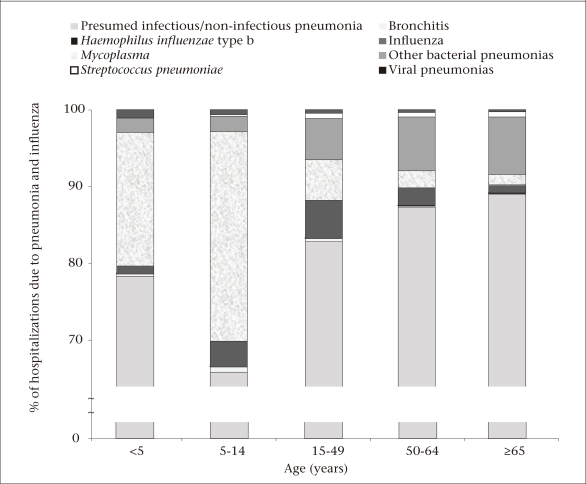
Percentage of hospitalizations due to pneumonia and influenza by diagnostic group and age-group, 2002-2005

Of all the pneumonia and influenza-associated hospitalizations, 48.4% (n=478,751) occurred among children aged less than five years. An additional 21.2% (n=208,143) occurred among persons aged 5-49 years, and 21.5% (n=212,615) occurred among the elderly aged 65 years and above. Among all the age-groups, males (55.9%) were more often hospitalized but this difference was most pronounced in the first three decades of life and in persons aged over 60 years (p<0.05). Most (81.3%; n=804,116) pneumonia and influenza-associated hospitalizations were coded with diagnoses with unspecified aetiology. Of these, 55.9% (n=448,986) occurred among children aged less than 15 years. Of all the hospitalizations due to pneumonia and influenza, 18.7% (n=185,356) were associated with aetiology-specific diagnoses. Of these, 64.2% (n=118,922) were associated with *M. pneumoniae,* 20.8% (n=38,563) with other pneumonia-causing bacteria, 9.1% (n=16,885) with influenza viruses, 3.9% (n=7,219) with other viruses that cause pneumonia, 1.8% (n=3,358) with *S. pneumoniae,* and 0.2% (n=409) with *H. influenzae*. *Mycoplasma* was frequently diagnosed among hospitalized children aged less than five years (17.3%) and children aged less than 15 years (27.2 %) while influenza was frequently diagnosed among adults aged 15-49 years (Table 2 and Fig. 1).

**Table 1.** Characteristics of hospitalizations and deaths due to pneumonia and influenza in Korea, 2002-2005

Year	Population covered by NHIC	All causes of hospitalizations	Hospitalizations due to pneumonia and influenza		All causes of death	Deaths due to pneumonia and influenza	
			No.	%		No.	%
2002	46,659,476	4,753,009	219,013	4.61	NA	NA	NA
2003	47,102,786	5,399,067	241,585	4.47	245,860	2,829	1.15
2004	47,371,992	5,706,573	254,285	4.46	245,795	3,522	1.43
2005	47,392,052	5,922,751	274,589	4.64	245,508	4,192	1.71

NA=Not available;

NHIC=National Health Insurance Corporation

The number of deaths due to pneumonia and influenza increased by 48.2% from 2,829 in 2003 to 3,522 in 2004 and 4,192 in 2005. Most (98.1%) deaths due to pneumonia and influenza were associated with presumed infectious pneumonia during 2003-2005 (2,777 in 2003; 3,459 in 2004; and 4,110 in 2005) while the number of deaths caused by influenza was very limited from 20 in 2003 to 38 in 2004 and 6 in 2005.

### Rates of hospitalizations and mortality

**Fig. 2.**
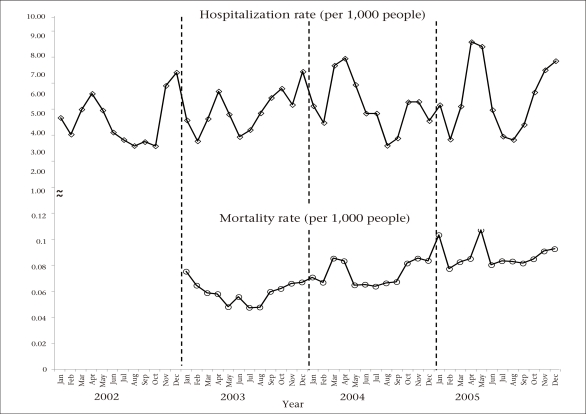
Monthly distribution of hospitalization rates, 2002-2005 and mortality rates, 2003-2005 due to pneumonia and influenza

During 2002-2005, the monthly rate of hospitalizations due to pneumonia and influenza increased significantly (p=0.038) (Fig. 2). A peak of hospitalizations due to pneumonia and influenza occurred in the final weeks of 2002, and two broader peaks occurred in the spring of 2004 and 2005. In contrast, the mortality rate declined in the first six months of 2003 and then increased steadily from 2003 through 2005.

**Fig. 3.**
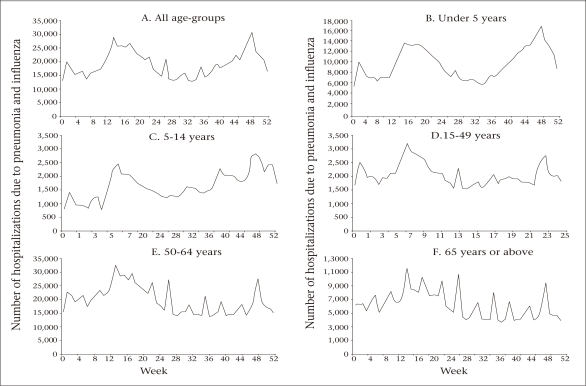
Weekly distribution of hospitalizations due to pneumonia and influenza by age-group, 2002-2005

The monthly distribution of hospitalizations due to pneumonia and influenza also varied by age-group. In the combined four-year database, the burden of diseases in children had two seasonal peaks (Fig. 3A and 3B)one during the autumn and winter months (October to January) and the second one during the spring (April to June). Hospitalizations among children aged 5-14 years (Fig. 3B and 3C) showed a winter seasonal peak with longer duration and a shorter spring peak than children aged less than five years. Young adults aged 15-49 years and middle-aged adults (50-64 years) (Fig. 3D and 3E) experienced smaller and shorter winter epidemics but the spring epidemics in these age-groups showed higher peaks and longer durations compared to the elderly. Persons aged 65 years and above (Fig. 3F) had a large, sustained year-round burden of hospitalizations due to pneumonia and influenza, with several smaller peaks and durations in all the years (p<0.05).

For all the hospitalizations due to pneumonia and influenza, the mean duration of stay was 9.6 patient-days (median 7 days). By age-group, the median duration (days) of stay increased with age from six days among children aged less than five years to 9.5 days in the elderly aged 65-74 years and 10 days among those aged ≥75 years (data not shown).

The average annual rate of hospitalizations due to pneumonia and influenza was 5.2 per 1,000 people (95% CI 5.2-5.3) in all ages during the study period. During 2002-2005, the overall rate of hospitalizations increased by 27.5% from 4.5 per 1,000 people to 5.8 per 1,000 people (Table 3). Children aged less than five years (37.5 per 1,000 people) and the elderly aged ≥75 years (20.3 per 1,000 people) had the highest age-group-specific hospitalization rates for pneumonia and influenza over the four-year study period. When we adjusted the hospitalization rate using the 2005 census data, children aged less than five years and the elderly aged ≥65 years showed the increased annual rates of hospitalizations due to pneumonia and influenza while young children and adults aged 5-49 years had the similar adjusted annual hospitalization rates during the four-year study period. The hospitalization rate among the elderly aged ≥75 years increased by 92.4% (from 13.4 to 25.7 per 1,000 people) and by 13.6% among children aged less than five years (from 43.5 to 49.4 per 1,000 people). As the age increased, the percentage of increase in the hospitalization rates over the study period rose dramatically from 30.2% among those aged 50-64 years to 51.1% for those aged 65-74 years and 92.4% for patients aged ≥75 years.

**Table 2.** Hospitalizations due to pneumonia and influenza by diagnosis and age-group, Korea, 2002-2005

Diagnosis	No. (%) of hospitalizations by age-group (years)						Total
	<5	5-14	15-49	50-64	65-74	≥75	
Aetiology unspecified							
Presumed infectious/non-infectious pneumonia	378,802 (47.3)	68,169 (8.5)	86,640 (10.8)	78,513 (9.8)	93,667 (11.7)	95,612 (11.9)	801,403
Bronchitis	1,379 (50.8)	636 (23.4)	396 (14.6)	155 (5.7)	89 (3.3)	58 (2.1)	2,713
Aetiology-specific							
*Haemophilus influenzae*	59 (14.4)	15 (3.7)	46 (11.2)	67 (16.4)	88 (21.5)	134 (32.8)	409
Influenza	4,597 (27.2)	2,896 (17.2)	5,228 (31.0)	2,060 (12.2)	1,356 (8.0)	748 (4.4)	16,885
*Mycoplasma pneumoniae*	79,503 (66.9)	28,791 (24.2)	5,596 (4.7)	2,011 (1.7)	1,692 (1.4)	1,329 (1.1)	118,922
*Streptococcus pneumoniae*	490 (14.6)	159 (4.7)	652 (19.4)	600 (17.9)	744 (22.2)	713 (21.2)	3,358
Other bacterial pneumonias	8,850 (22.9)	2,033 (5.3)	5,666 (14.7)	6,262 (16.2)	7,805 (20.2)	7,947 (20.6)	38,563
Other viral pneumonias	5,071 (70.2)	707 (9.8)	513 (7.1)	295 (4.1)	333 (4.6)	300 (4.2)	7,219
All pneumonias and influenzas	478,751 (48.4)	103,406 (10.5)	104,737 (10.6)	89,963 (9.1)	105,774 (10.7)	106,841 (10.8)	989,472

## DISCUSSION

In Korea, the increased number and rate of hospitalizations due to pneumonia and influenza and the increased number of deaths in all age-groups were observed despite the introduction of pneumococcal vaccine in 2003 and the introduction of influenza vaccine in 1997 (17, 18). In addition, rates of hospitalizations due to pneumonia and influenza and mortality were high among very young and elderly persons. The burden of hospitalizations due to pneumonia and influenza among the elderly has increased sharply with the ageing of the Korean population. Over the four-year study period, the rate of hospitalizations due to pneumonia and influenza among those aged 75 years and above increased by 92.4%. From a national perspective, the dramatic rise in the rates of hospitalizations due to pneumonia and influenza is of concern as the population in this age-group increased by 19% during the same period. The NHIC reported that the total cost of hospitalizations for the elderly aged 65 years or above increased faster (62%) than the total number of hospitalizations (19%) nationwide over the four-year period from 2002 to 2005 (19). These demographic trends observed in Korea mirror those found in other developed nations of Asia and Europe (20). Nevertheless, population shifts alone are unlikely to entirely explain the rise in hospitalizations due to pneumonia and influenza. Given the recent trends in diseases, it is likely that a substantial proportion of hospitalizations due to pneumonia and influenza in the elderly occurred among those with the underlying medical conditions. It has been particularly recognized that chronic medical conditions may place the elderly at a higher risk for serious pneumonia and influenza (15,21). In 2005, Korea spent 6% of its gross domestic product (GDP) on healthcare, one-third less than the 9% of GDP health expenditure averaged among 30 economically-developed nations (22). Nevertheless, the increasing burden of hospitalizations due to pneumonia and influenza in Korea underscores the massive costs of these diseases to society and the healthcare system. These observations suggest that public-health strategies to prevent pneumonia and influenza are likely to be cost-effective in Korea.

A few comparable national studies were conducted that allow for age-group comparisons. Elsewhere, there is a strong evidence that hospitalizations due to pneumonia and influenza occur at high rates. For example, in Spain, the annual hospitalizations due to pneumonia and influenza among the people aged 50-64 years occured in 143 cases per 100,000 people (23). In England, the annual hospitalizations due to pneumonia episodes ranged from 10 to 15 per 1,000 people, with the hospital admission rates of 1-4 per 1,000 people per year among children aged ≤5 years (24). A study of all-cause community-acquired pneumonia among patients aged above 14 years in Spain reported an overall hospitalization rate of 1.62 per 1,000 people (25). In 2005, Burgner and Richmond reported a pneumonia-related hospitalization rate of 5-8 per 1,000 person-years among Australian children aged less than five years (26). Results of an analysis of the US Medicare insurance records showed a high rate of hospitalizations due to pneumonia and influenza among the elderly (15.1-23.4 per 1,000 people), with a 55% increase in hospitalization over a 13-year period (27). We also found that children aged less than five years had the highest age-specific rate (37.5 per 1,000) of hospitalizations due to pneumonia and influenza among all age-groups. In our study, the hospitalizations rate for childhood pneumonia was higher compared to the mean annual excess influenza-associated hospitalizations among Hong Kong children (1999 data: <1-year old, 28.8 per 1,000; 1-2-year old, 20.9 per 1,000; and 2-5-year old, 7.7 per 1,000 children) (28). We also found that hospitalizations due to pneumonia and influenza were distributed with the distinct winter and spring peaks in all age-groups. These classical winter and spring peaks of pneumonia and influenza are consistent with data from Australia (26), Spain (29), the USA (30,31), Brazil (32), and Viet Nam (33). The year-to-year variations observed in the seasonal pneumonia and influenza-associated hospitalization peaks may be due to annual variations in the circulating influenza virus subtypes or other pathogens found in Korea. The laboratory-based national influenza surveillance system reported predominantly H3N2 influenza virus isolates in the 2002-2003, 2003-2004 and 2004-2005 influenza seasons, which likely influenced the seasonal peak distribution observed in this analysis. The pneumonia and influenza-associated hospitalization peak that started in the winter of 2005 was larger than previous years and was associated with a new influenza subtype H1N1 (34).

**Table 3.** Annual pneumonia and influenza-associated hospitalizations by year and age-group, Korea, 2002-2005

Age-group (years)	Annual hospitalizations rates*											Rate change (%)†
	2002			2003			2004			2005		
	No. (%)	Unadjusted	Adjusted	No. (%)	Unadjusted	Adjusted	No. (%)	Unadjusted	Adjusted	No. (%)	Rate	
<5	110,645 (50.5)	36.7	43.5	117,841 (48.8)	41.2	46.3	124,510 (49.0)	45.8	48.9	125,755 (45.8)	49.4	13.6
5-14	21,924 (10.0)	3.3	3.3	33,607 (13.9)	5	5.0	21,581 (8.5)	3.2	3.2	26,294 (9.6)	3.9	19.1
15-49	25,640 (11.7)	0.9	0.9	26,210 (10.8)	0.9	1.0	25,664 (10.1)	0.9	0.9	27,223 (9.9)	1.0	7.1
50-64	20,079 (9.2)	3.1	2.8	19,785 (8.2)	3	2.8	24,074 (9.5)	3.5	3.4	26,025 (9.5)	3.7	30.2
65-74	21,520 (9.8)	8.4	7.3	22,658 (9.4)	8.4	7.7	29,193 (11.5)	10.4	10.0	32,403 (11.8)	11.1	51.1
≥75	19,205 (8.8)	15.9	13.4	21,484 (8.9)	16.9	14.9	29,263 (11.5)	21.6	20.4	36,889 (13.4)	25.7	92.4
Total	219,013 (100)	4.6	4.5	241,585 (100)	5.1	5.0	254,285 (100)	5.3	5.3	274,589 (100)	5.8	27.5

*Annual hospitalization rate per 1,000 people; adjusted for the population in 2005 census data;

†Rate change (%)={rate (2005)-adjusted rate (2002)}/adjusted rate (2002)*100

### Limitations

This study has some limitations. As with other studies of administrative hospitalization databases, the potential for diagnostic misclassification must be considered. In this study, we were unable to identify individual patients and then conduct a validation of diagnoses in the hospital patient-charts. Nonetheless, it is unlikely that patients who presented with signs and symptoms of pneumonia and influenza were misclassified into non-pneumonia and influenza diagnostic categories given the distinct clinical nature of lower respiratory tract disease (35-37). We recognize that our analysis may include patients with nosocomially-acquired pneumonia and influenza. To reduce the potential for including nosocomial pneumonia, we removed records where the date of diagnosis occurred after the date of admission prior to our final analysis. In addition, we did not evaluate cohort effects that may influence age-and time-related patterns of pneumonia and influenza using regression analysis (7). Such analysis will be considered in future analysis that will include additional years of data. In future analysis, laboratory-confirmed diagnoses obtained from the national surveillance systems may be linked to hospitalization records in the national health-insurance databases to validate ICD-10 diagnoses. In addition, we recommend further studies of the burden of pneumonia and influenza in Korea that may begin by comparing hospitalizations before and after the introduction of pneumococcal and influenza vaccines into the national immunization programmes.

### Conclusions

The growing toll of hospitalizations due to pneumonia and influenza in Korea is of concern. Such trends underscore the need for a comprehensive review of preventive health services that may reduce the burden of pneumonia and influenza. This review of services in Korea should consider prospective evaluation of the impact and cost-effectiveness of vaccination. Given the epidemiologic profile of hospitalizations due to pneumonia and influenza in these national data, it is likely that health policies focused on the prevention of pneumonia and influenza will provide important opportunities for reducing the total burden of pneumonia and influenza among the Korean population.

## ACKNOWLEDGEMENTS

The authors thank I.S. Park, Division of Statistical Analysis, National Insurance Corporation Research Institute, for technical support and assistance for data management. Robert Holman, Office of Director, Division of Viral and Rickettsial Diseases, National Center for Infectious Diseases, Centers for Disease Control and Prevention, assisted with data analysis, interpretation, and review of the manuscript. They also thank Kathy Murray, a freelance technical editor, for editorial assistance.
